# Thyroid Stimulatory Activity of *Houttuynia cordata* Thunb. Ethanolic Extract in 6-Propyl-Thiouracil-Induced Hypothyroid and STZ Induced Diabetes Rats: In Vivo and In Silico Studies

**DOI:** 10.3390/nu17030594

**Published:** 2025-02-06

**Authors:** Shaikh Shahinur Rahman, Anuwatchakij Klamrak, Nirmal Chandra Mahat, Rakibul Hasan Rahat, Napapuch Nopkuesuk, Md Kamruzzaman, Piyapon Janpan, Yutthakan Saengkun, Jaran Nabnueangsap, Thananya Soonkum, Padol Sangkudruea, Nisachon Jangpromma, Sirinan Kulchat, Rina Patramanon, Arunrat Chaveerach, Jureerut Daduang, Sakda Daduang

**Affiliations:** 1 Division of Pharmacognosy and Toxicology, Faculty of Pharmaceutical Sciences, Khon Kaen University, Khon Kaen 40002, Thailand; shahin@anft.iu.ac.bd (S.S.R.); anuwat_kla@yahoo.com (A.K.); napapuch.aom25@gmail.com (N.N.); j.piyapon@kkumail.com (P.J.); yutthakan_s@kkumail.com (Y.S.); 2 Department of Applied Nutrition and Food Technology, Faculty of Biological Sciences, Islamic University, Kushtia 7000, Bangladesh; nirmal.anft.iu@gmail.com (N.C.M.); rakibulrahat5@gmail.com (R.H.R.); mkzaman.m@gmail.com (M.K.); 3 Salaya Central Instrument Faculty RSPG, Research Management and Development Division, Mahidol University, Nakhon Pathom 73170, Thailand; jaran.nab@mahidol.edu (J.N.); thananya.soo@mahidol.edu (T.S.); 4 Success Time Enterprise Co., Ltd., 194/3 Moo 9 Tumbol Muang Kao, Amphor Muang, Khon Kaen 40000, Thailand; padol2513@gmail.com; 5 Faculty of Science, Khon Kaen University, Khon Kaen 40002, Thailand; nisaja@kku.ac.th (N.J.); sirikul@kku.ac.th (S.K.); narin@kku.ac.th (R.P.); raccha@kku.ac.th (A.C.); 6 Protein and Proteomics Research Center for Commercial and Industrial Purposes (ProCCI), Khon Kaen University, Khon Kaen 40002, Thailand; 7 Department of Clinical Chemistry, Faculty of Associated Medical Sciences, Khon Kaen University, Khon Kaen 40002, Thailand; jurpoo@kku.ac.th

**Keywords:** hypothyroidism, diabetes, *Houttuynia cordata*, molecular docking, phytochemical profiling, cheminformatics

## Abstract

*Houttuynia cordata* Thunb. holds a longstanding reputation as a traditional folk remedy in East Asia, where it has been employed to treat a variety of inflammatory conditions, nephritis, hepatitis and cancer. Despite its extensive use, there exists a paucity of research examining its efficacy in managing thyroid disorders and diabetes. Moreover, the bioactive components responsible for modulating the molecular pathways remain elusive. Objectives: This research aimed to determine the key bioactive components in the ethanolic extract of *H. cordata* Thunb. (HCEE) responsible for its thyroid-modifying properties and examine its effects on rats with experimentally induced hypothyroidism and diabetes. Methods: Molecular docking was performed to investigate the possible mechanisms of thyroid regulation of HCEE constituents. Researchers induced hypothyroidism in rats by adding 6-propyl-2-thiouracil to their drinking water for a period of four weeks. To induce diabetes, the rats received an intraperitoneal injection of streptozotocin. The animals were then given daily oral doses of HCEE (500 mg/kg b.w.), levothyroxine (50 mg/kg b.w.), or glibenclamide (5 mg/kg b.w.) for 28 days. Following this treatment, standard methods were employed to measure biochemical parameters in the rats’ serum. Results: The results demonstrate that HCEE ameliorated hypothyroidism by increasing serum T3 (14.38%) and T4 (125.96%) levels and decreasing TSH (*p* < 0.01; −41.75%) levels. In diabetic rats with induced hypothyroidism, HCEE significantly (*p* < 0.001) increased T3 (149.51%) and T4 (73.54%) levels with reduced TSH (−64.39%) levels. In silico analysis demonstrated that the identified bioactive compounds from HCEE may enhance thyroid hormone function through interaction with the thyroid hormone receptor protein TRβ1 (PDB:3GWS), similar to the conventional pharmaceuticals levothyroxine and triiodothyronine (T3). Conclusions: HCEE exhibits potential as a natural alternative to synthetic medications in the prevention and treatment of thyroid dysfunctions.

## 1. Introduction

Endocrine disorders such as diabetes and thyroid problems often coexist and can negatively impact each other [1]. Unmanaged thyroid conditions can disrupt metabolic regulation and elevate the likelihood of diabetic patients developing additional health complications [2]. Thyroid hormones have a direct influence on insulin secretion and can increase hepatic glucose output by upregulating gene expression of glucose transporters (GLUT2, GLUT4) and phosphoglycerate kinase [3]. Thyroid disorders affect insulin function differently: hypothyroidism reduces β-cell insulin sectretion in response to glucose, while hyperthyroidism increases insulin resistance. Globally, an estimated 300 million people are affected by thyroid disorders, which are more prevalent in diabetic patients and are considered the second most frequent endocrine disorder [4]. The prevalence of thyroid disorders is 6.6%, with hypothyroidism being the most common autoimmune thyroid condition, increasing in frequency with age. Recent studies show that women have a higher rate of hypothyroidism (15.86%) compared to men (5.02%) [5]. Furthermore, one in eight women faces the risk of developing a thyroid disease during their lifetime [6]. In hypothyroidism, thyroid hormones are below normal levels. This can result in symptoms like fatigue, cold intolerance, weight gain, and chronic constipation [7]. Untreated hypothyroidism can result in metabolic disorders, including diabetes mellitus and cardiovascular diseases. Notably, approximately 17–30% of diabetic patients exhibit a prevalence of thyroid disorders [2]. Individual with subclinical hypothyroidism faces an elevated likelihood of symptomatic hypoglycemia. This increased risk stems from several factors: diminished glucose uptake in the digestive symtem, reduced glucose production by the liver, and decreased synthesis of new glucose molecules [8]. In the case of severe hypothyroidism, counter-regulatory hormones, viz. glucagon, epinephrine, and cortisol, are increased in levels [9]. Multiple studies have shown that hypothyroidism is linked to a hypometabolic condition, which may result in decreased production of free radicals and a reduction oxidative damage [10,11]. Conversely, diabetes is characterized by pathological complications caused by the opposite effect. Additionally, in individuals with hypothyroidism often experience various abnormalities in lipid metabolism, including dyslipidemia and an increased risk of cardiovascular diseases [12,13]. Hypothyroidism is usually treated with levothyroxine (T4). However, long-term use of T4 can lead to side effects like cardiac dysfunction, left ventricular hypertrophy, and liver damage related to oxidative stress [14]. Therefore, developing a new therapy that can improve thyroid function without causing any or minimal side effects is essential. Rahman et al. (2021) argue that plant-based medicines help treat various diseases with negligible or no side effects due to the presence of many phytocompounds that are biologically active against diseases [15].

Plant-derived compounds known as phytochemicals can exert both beneficial and detrimental effects on the thyroid gland’s functionality. Numerous flavonoids, including but not limited to myricetin, quercetin, apigenin, rutin, genistein, curcumin, resveratrol, and catechins, have the potential to influence the expression and activity of various enzymes, proteins, transporters, and receptors, these components play crucial roles in the production, movement, breakdown, and elimination of thyroid hormones, as well as their activation within the cell [16]. However, some flavonoids and isoflavones, like phytoestrogens, genistein, and daidzein, can cause endemic goiter and hypothyroidism [17]. As a result, there is growing interest in identifying appropriate plants with potential phytochemical effects on thyroid function, which could offer possible advantages for conditions such as hypothyroidism and diabetes or associated disorders.

*Houttuynia cordata* Thunb., a member of the Saururaceae family, is a plant with medicinal properties. In Thailand it is referred to as ‘Khaotong’ or ‘Plu kaow’. This plant species ir prevalent throughout East Asia. The plant has been used as a folk medicine for various purposes and is often used to prepare fermented beverages, nutraceuticals, feed, and cosmetics [18]. This plant is traditionally consumed for treating diabetes, constipation, hyperlipidemia, neural problems, and liver diseases due to its antioxidative, hypolipidemic, and acetylcholinesterase inhibitory properties [19,20]. It exhibits potent antidiabetic properties by stimulating insulin secretion, regenerating pancreatic beta cells, and inhibiting glucose absorption, as shown in previous studies [21]. However, the potential effects of *H. cordata* ethanolic extract (HCEE) on thyroid function have not yet been investigated. Some studies suggest that pentacyclic triterpenes, such as betulinic acid, lupeol, betuline, oleanolic acid, alpha, and beta amyrins, can help alleviate hypothyroidism [22]. This is because they prevent NF-kb activation, which is known to interfere with the T3-dependent induction of 5′-Deiodinase gene expression. This interference can lead to a reduction in thyroid hormone production [23]. Kahksha et al. (2023) report that HCEE regulates cellular metabolism, gene expression, and signaling pathways [24]. Taking into account the aforementioned information, we postulated that HCEE could have a favorable influence on hypothyroidism. This research constitutes the initial attempt to investigate the consequences of HCEE on hypothyroidism. Therefore, the main aim of this research is to determine if this plant extract can mitigate the symptoms of hypothyroidism in a rats with PTU-induced hypothyroidism, along with related conditions.

## 2. Materials and Methods

### 2.1. Plant Collection and Preparation of H. cordata Extract

Researcher obtained a fresh sample of aerial *H. cordata* plants from an open field in Than Thong, Phan District, Chiang Rai, Thailand (19°42′34.9″ N 99°41′28.5″ E). The plant components (leaves, stalks, and trunks) were subjected to thorough cleaning in running water for 30 to 45 min to eliminate undesired residue. Follow the cleaning process, the plant samples were desiccated at 50 °C for two to three days using in an oven incubator (BINDER Inc., Bohemia, NY, USA) and subsequently pulverized using a blender.

To prepare the ethanolic extract, 20 g of the finely ground material was combined with 200 mL of ethanol and agitated at 25 °C for two days using an incubator shaker (N-BIOTEK, Bucheon, Gyeonggi, Republic of Korea). The resulting clear supernatant was obtained through centrifugation at 8000 rpm for 10 min at 25 °C and transferred to a round bottom flask for rotary evaporation at 45 °C for one hour. The samples were then subjected to lyophilization (BUCHI, Rotavapor, Roskilde, Denmark) at −110 °C for 48 h and stored at −80 °C until required for use.

### 2.2. Chemicals and Reagents

All chemicals, reagents, and biochemical kits were of analytical grade and were obtained from Sigma-Aldrich (St. Louis, MO, USA). Ethanol (99.9% pure) was procured from Merck, Darmstadt, Germany. Serum biochemical parameters were measured in accordance with the manufacturer’s instructions utilizing an automatic analyzer (HumaStar 300) (Human GmbH, Wiesbaden, Germany). The materials were stored at 4 °C.

### 2.3. In Silico Analysis

To elucidate the potential mechanism underlying the thyroid stimulatory activity of the active compounds in HCEE, we conducted molecular docking studies to analyze the possible interactions between isolated compounds from HCEE and the target protein, thyroid hormone receptor β (TRβ1). The PDB file for TRβ1 protein, with a resolution of 2.20 Å, was obtained from the Protein Data Bank (PDB ID: 3GWS) at https://www.rcsb.org/structure/3GWS (accessed on 4 September 2024). We utilized the CB-Dock2 server (accessed on 4 September 2024 at https://cadd.labshare.cn/cb-dock2/php/blinddock.php#job_list_load) to predict the binding poses and generate complex structures of TRβ protein with each HCEE metabolite. This molecular docking tool employs AutoDock Vina (v.1.2.0) during the docking process and provides the negative Gibbs free energy values for the receptor-ligand complexes with acceptable RMSD. The interaction of the predicted interactions of TRβ-HCEE complex was identified and visualized using the BIOVIA Discovery Studio 2021 Client software. The drug-likeness, pharmacokinetics, and physicochemical properties of HCEE metabolites were analyzed via SwissADME web server (http://www.swissadme.ch/).

### 2.4. Animals

Male Long Evans rats (150–230 g) were obtained from the animal facility of the Faculty of Biological Sciences, Islamic University, Bangladesh. The rats were allowed a one-week acclimatization period prior to the commencement of the experiment. During this interval, the rats were provided with standard laboratory chow and ad libitum access to drinking water. Throughout the experimental period, standard environmental conditions were maintained, including a temperature of 24 ± 2 °C, relative humidity of 45 ± 5%, and a 12-h light-dark cycle.

### 2.5. Diet for the Animals

All the groups of rats were fed a daily diet that was recommended by Rahman et al. (2021a) [25]. This diet included 30% wheat flour, 10% fishmeal as a protein source, 20% rice polish, 10% oilseed cake, 21% wheat bran, 5% molasses, 0.5% vitamins, 1.5% common salt and 2% soybean oil, which is regarded as a standard laboratory diet.

### 2.6. Experimental Design

We divided the rats into nine groups (*n* = 8) to investigate how HCEE affected hypothyroidism and hyperglycemia:

Group 1: Untreated healthy control rats (HC)
Group 2: Untreated hypothyroid control rats (0.05% 6-propyl-2-thiouracil was given in drinking water for 4 weeks) (HTC)
Group 3: Untreated diabetic control rats (65 mg/kg b.w. streptozotocin injected intraperitoneally) (DC)
Group 4: Untreated diabetic with hypothyroid control rats (DHTC)
Group 5: Healthy rats treated with HCEE (500 mg/kg/day in 1 mL water) (HHCEE)
Group 6: Hypothyroid rats treated with HCEE (500 mg/kg/day in 1 mL water) (HTHCEE)
Group 7: Hypothyroid rats treated with levothyroxine (50 mg/kg b.w./day in 1 mL water) (HTD)
Group 8: Diabetic rats treated with HCEE (500 mg/kg/day in 1 mL water) (DHCEE)
Group 9: Diabetic rats treated with glibenclamide (5 mg/kg/day in 1 mL water) (DD)

An experiment was performed utilizing HCEE at a dose of 500 mg/kg/rat/day. The extract was suspended in 1 mL of distilled water and orally administered to rats in groups 5, 6, and 8, alongside their standard diet. Concurrently, the control groups consisting of untreated healthy control, hypothyroid, and diabetic control rats (groups 1, 2, 3, and 4) were provided with drinking water. Groups 7 and 9 received reference drugs: L-thyroxine (50 mg/kg b.w./day) and glibenclamide (5 mg/kg/day). The study lasted 28 consecutive days, with regular monitoring and documentation of progress.

### 2.7. Induction of Hypothyroidism and Diabetes in Rats

To induce hypothyroidism in the experimental rats, a well-established method [26] was employed, involving the administration of 0.05% 6-propyl-2-thiouracil (PTU) in drinking water for a period of 4 weeks. Rats were classified as hypothyroid when their values exceeded the normal ranges (TSH: 0.4–4.5 mU/L; FT4: 19–25.60 pmol/L; and FT3: 3.20–9.20 pmol/L) [27]. Diabetes was induced by administrating fasted rats intraperitoneally with a single dose of freshly prepared streptozotocin (STZ) (Sigma, St. Louis, MO, USA) at 65 mg/kg body weight, dissolved in 0.1 M cold sodium citrate buffer (pH of 4.5) [28]. Blood glucose levels were measured in fasted rats three days after STZ administration, following beta cells were destrustion Rats with fasting blood glucose (FBG) levels exceeding 250 mg/dL (>13.8 mmol/L) were selected for the experiments to confirm their diabetic status [29]. Control and experimental rats were housed separately in metabolic cages.

### 2.8. Blood Collection and Biochemical Parameters Determination

Weekly blood samples were obtained from the animals’ tail veins weekly following a 10–12 h fasting period. Glucose levels were measured using a glucometer (Accu Check Advantage^®^, Roche Diagnostics, Mannheim, Germany) with glucose oxidase/peroxidase reactive strips. Upon completion of the treatment, the animals were subjected to overnight fasting, and blood was collected from their retro-orbital plexus. The blood samples were centrifuged at 3000 rpm for 10 min to separate the serum and were subsequently stored at −80 °C until analysis.

Rat-specific ELISA kits from Cusabio Biotech Co., Ltd., Wuhan, China were employed to determine serum T3, T4, and TSH levels following standard methods [30]. The manufacturer specified that these kits had an intra-assay and inter-assay precision of less than 15%. T3 and T4 measurements utilized a competitive inhibition enzyme immunoassay technique. For T3 measurements, samples or standard were added to microtiter plates coated with T3 specific antibodies, followed by biotin-conjugated T3. The biotin-conjugated T3 competed with the T3 in the sample or standard for antibody binding. After washing, avidin-conjugated horseradish peroxidase (HRP) was added, followed by the substrate. The resulting color intensity was inversely proportional to the T3 concentration in the sample/standard. T4 measurement followed a similar process, with T4 specific antibody-coated plates and and biotin-conjugated T4. TSH was measured using a quantitative sandwich enzyme immunoassay technique. TSH-specific antibody-precoated microtiter plates were utilized, and standards and samples were added to the well along with HRP-conjugated TSH-specific antibodies. Following a wash step, the substrate was introduced, and the color intensity developed was directly proportional to the TSH concentration in the sample/standard.

### 2.9. Statistical Analysis

SPSS version 24 (SPSS/IBM, Chicago, IL, USA) was employed for the statistical analysis, with ANOVA utilized for each group. Paired or un-paired *t*-tests were used to conduct multiple comparisons. The findings were expressed as mean ± standard deviation (SD). Statistical significance was defined as *p* < 0.05, while *p* < 0.001 was considered highly significant.

## 3. Results

### 3.1. Molecular Docking

The potential mechanism underlying the reversal of hypothyroidism is complex, involving multiple pathways and target proteins. The specific target receptor protein, TRβ1 (PDB:3GWS), is a member of the nuclear receptor superfamily and serves as an exemplary model of cellular signaling due to its activation by diverse ligands. Thyroid hormone receptors (TRs) function as ligand-dependent transcription factors that regulate gene transcription through activation or repression, thereby modulating numerous physiological processes. While 3,3,5-L-triiodothyronine (T3) is the natural ligand for TRs, other substances can also activate these receptors. These alternative ligands, which can be either natural or synthetic, have the ability to induce different active conformations in the receptors, thereby influencing the signaling pathways related to thyroid hormone activity [31].

To identify potential bioactive components that stimulate the thyroid in the HCEE, we conducted LC-MS/MS analyses. This is because the major components present in an extract play a crucial role in its biological activity. A recent study confirmed the identification, characterization, and quantification of bioactive compounds from plu kaow (*H. cordata*) using the advanced method UPLC–ESI–QqQLIT–MS/MS [32]. As a result, we aimed to investigate these significant compounds of HCEE through molecular docking studies with a critical thyroid hormone receptor: thyroid hormone receptor β (TRβ) (PDB:3GWS). Considering that TRβ’s established biological function is to regulate the synthesis and secretion of thyroid hormones [33], we examined the interaction of these HCEE components with this receptor.

**Figure 1:**
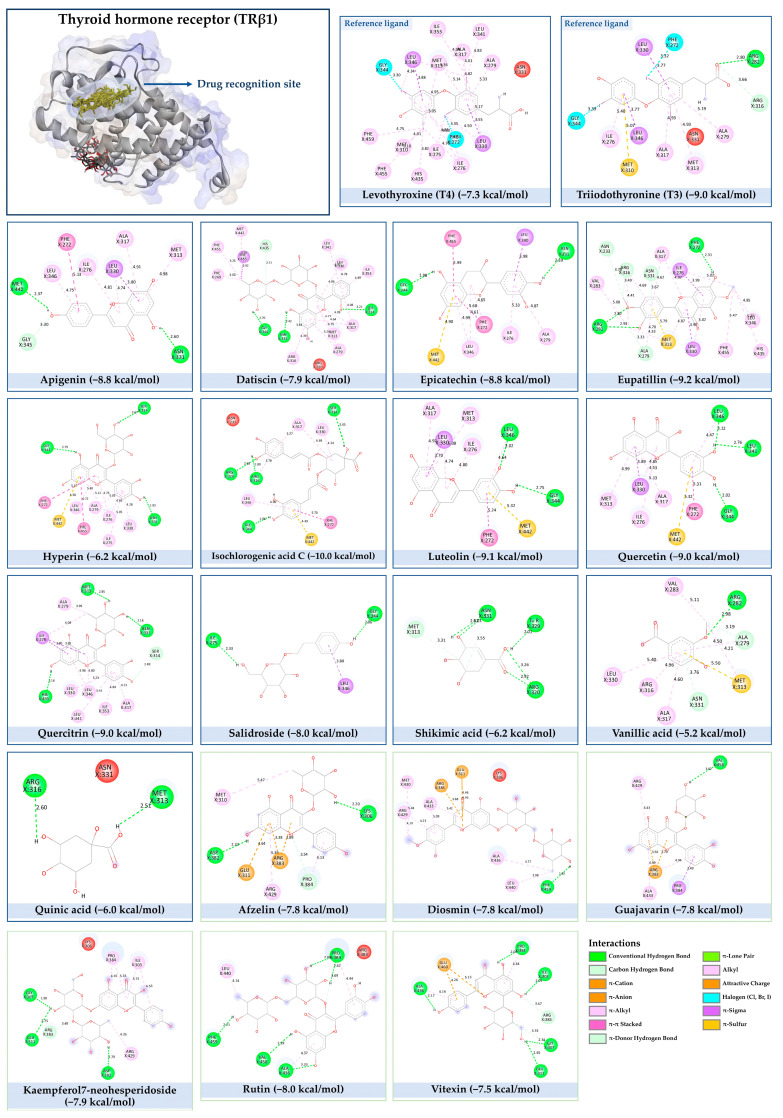
Bioactive compounds from *H. cordata* ethanolic extract showed 3D and 2D representation of the interaction of levothyroxine (T4), triiodothyronine (T3), eupatilin, luteolin, isochlorogenic acid C, shikimic acid, quercetin, quercitrin, epicatechin, vitexin, apigenin, rutin, salidroside, kaempferol 7-neohesperidoside, datiscin, afzelin, diosmin, guaijaverin, hyperin, quinic acid, and vanillic acid with thyroid hormone receptor protein (3GWS).

Our comprehensive metabolomics investigation and computational analyses have thoroughly examined HCEE and revealing numerous flavonoids and other glycosylated derivatives. We investigated the molecular mechanisms underlying the anti-hypothyroidism effects of specific simple phenolics, flavonoids, and their glycosylated counterparts in rat models (as shown in Table 1). Our study incorporated molecular docking analyses of the PDB:3GWS receptor. The in-silico evaluation suggested that the examined ligands are likely to demonstrate anti-hypothyroid properties by interacting with multiple receptors, exhibiting binding energies surpassing those of conventional anti-hypothyroid medications such as levothyroxine. Several compounds, including eupatillin, luteolin, quercetin, quercitrin, epicatechin, apigenin, rutin, salidroside, kaempferol 7-neohesperidoside, isochlorogenic acid C, datiscin, afzelin, diosmin, and guaijaverin, displayed robust interactions with all tested receptors, highlighting the polypharmacological potential of these phenolic compounds. This study also indicated that certain natural compounds, including isochlorogenic acid C, eupatilin, luteolin, quercetin, and quercitrin, demonstrated potent binding energies (>−9.0 kcal/mol) [32]. These values differed marginally from those of conventional anti-hypothyroid medications such as levothyroxine (binding energy −7.3 kcal/mol), suggesting the potential efficacy of these natural substances. For a compound to be considered an active agent, it must specifically interact with the active site and drug recognition area of each receptor. This interaction should involve the formation of hydrogen and electrostatic bonds, with distances maintained within 3.00 Å and 5.00 Å, respectively [34]. It is hypothesized that the flavonoids found in the HCEE can regulate hypothyroid conditions by interacting with specific targets simultaneously. Compounds with high gastrointestinal absorption (GIA) and bioavailability scores were selected for docking investigations. The binding sites of compounds found in HCEE were identified through molecular docking analyses of the target protein TRβ1 (PDB:3GWS). The interaction between the ligands and the protein exhibited notable binding energies, as shown in Table 1 and Figure 1. Our research focused on examining the interactions between the primary polyphenols of HCEE and the TRβ1 (PDB:3GWS) protein, which are crucial targets for developing thyroid agonists. We compared the results with levothyroxine (T4) and triiodothyronine (T3) as positive controls.

#### 3.1.1. TRβ1 (PDB:3GWS)

As per the criteria, fifteen out of the 19 docked metabolites demonstrated activity owing to their stronger binding affinity to the TRβ1 protein in comparison to the reference drug levothyroxine (T4), which exhibited a binding energy of −7.3 kcal/mol. Notably, five metabolites—isochlorogenic acid C (−10.0 kcal/mol), eupatilin (−9.2 kcal/mol), luteolin (−9.1), quercetin (−9.0), and quercitrin (−9.0)—showed higher binding affinities towards TRβ1 than T3 (triiodothyronine), which had a binding energy of −9.0 kcal/mol. The remaining metabolites displayed varying binding affinities within the range of −5.2 to −8.8 kcal/mol (Table 1). The positive hypothyroidism control agent, triiodothyronine (T3), formed a hydrogen bond interaction with Arg282 (4.91 Å) in the TRβ1 structure and weakly formed a carbon-hydrogen bond interaction with Arg316 (3.96 Å). Additionally, it interacted with the active portion of the TRβ1 protein through various hydrophobic bond (π-alkyl, π-sulfur, and π-sigma) interactions involving specific residues Ile276, Ala317, Met313, Ala279, Met310, Leu330, and Leu346 at distances of 4.28 and 7.09 Å. On the other hand, levothyroxine (T4) did not show any hydrogen bond interactions with TRβ1. Instead, it exhibited π-alkyl, π-sigma, and alkyl interactions with active residues including Phe459, Phe455, Met310, His435, Ile275, Ile276, Ala279, Leu341, Ala317, Ile353, Met313, Leu330, and Leu346, with bond lengths ranging between 4.48 and 6.17 Å. Both levothyroxine and T3 showed halogen bond interactions with specific residues in the active site of the TRβ1 protein (Table 1).

Isochlorogenic acid C exhibited the highest binding affinity (−10.0 kcal/mol) among the compounds investigated, forming four conventional hydrogen bonds (1.89–3.05 Å) with Ser314, Arg316, Arg320, and Gly344 residues in the TRβ1 binding sites. This compounds also demonstrated hydrophobic interactions (3.78–5.27 Å), including alkyl, π-alkyl, and π-sulfur bonds, with proximal amino acids Arg316, Ala317, Leu330, Leu346, and Met442. The polyphenol interactions with key amino acid, Arg282, Arg320, and Asn331 likely contributed to its high negative binding energy.

Eupatilin (−9.2 kcal/mol) established three conventional hydrogen bonds (4.46–5.83 Å) with polar amino acids, such as Arg282, and Phe272, in the the target TRβ1 protein. Its aromatic system-attached methoxy group (-OCH_3_) was further stabilized through various hydrophobic bonds, including alkyl, π-alkyl, π-sigma, and π-sulfur interactions with Leu330, Ile276, Met313, Val283, Ala317, Leu346, His435, and Phe455 (4.39–6.97 Å). The compound’s sugar moiety formed three C-H interactions (3.93–5.52 Å) with Arg316, Asn233, and Ala279, as well as one π-donor hydrogen bond interaction with Asn331 (5.37 Å).

In comparison to T3 and levothyroxine, luteolin formed two strong conventional hydrogen bonds with Leu346 (3.48 Å) and Gly344 (4.49 Å). Its flavone skeleton facilitated hydrogen bond formation with six amino acid residues: Leu330, Phe272, Met442, Ala317, Met313, and Ile276 (ranging from 4.48 to 9.03 Å).

Quercitrin and quercetin exhibited identical binding energies (−9.0 kcal/mol) to T3. Quercitrin established three conventional hydrogen bond interactions with Asn331, Met313, and His435 (3.16–4.56 Å), in the TRβ1 structure and a weak carbon-hydrogen bond interaction with Ser314 (3.12 Å). It also interacted with the TRβ1 protein’s active region through various hydrophobic bond involving amino acid residues Ile276, Ala279, Ala317, Ile353, Leu341, Leu330, and Leu346, with bond lengths ranging from 4.23–6.64 Å.

Quercetin formed three strong hydrogen bonds with Leu341, Gly344, and Leu346 (3.63–4.17 Å). It demonstrated the capacity to engage in numerous π-alkyl, π-sigma, π-sulfur, and π-stacked interactions with eight amino acid residues, including Leu330, Phe272, Ala317, Ile276, Met313, Leu346, and Met442 (ranging from 4.72 to 9.00 Å).

Epicatechin and apigenin also displayed similar binding affinities (−8.8 kcal/mol), exceeding that of the reference drug (levothyroxine).

Within the active site of the target TRβ1 protein target, amino acids formed two conventional hydrogen bonds with Gly344 (1.98 Å) and Asn331 (2.39 Å), and as well as with Asn331 (4.21 Å) and Met442 (4.77 Å). Apigenin also exhibited a C-H bond interaction with Gly345 (3.57 Å) in the target area. It further developed various hydrophobic interactions with Phe272, Leu330, Ala317, Met313, Ile276, and Leu346 (4.44–6.98 Å). Epicatechin established nine alkyl, π-alkyl, π-sigma, π-sulfur, and π-stacked interactions with Leu380, Phe272, Ala279, Met442, Ile276, Leu346, and Phe455 (ranging from 3.98 to 5.99 Å).

Notably, rutin, salidroside, kaempferol 7-neohesperidoside, datiscin, afzelin, diosmin, guaijaverin, and vitexin demonstrated superior binding scores compared to the reference drug, levothyroxine (−7.3 kcal/mol) as shown in Table 1. The current study revealed that these investing metabolites formed conventional hydrogen bonds with varying bond lengths: 5 (2.94–6.12 Å), 2 (3.68–3.76 Å), 3 (3.51–4.92 Å), 3 (3.18–4.00 Å), 2 (3.71–3.93 Å), 1 (4.11 Å), 1 (5.94 Å), and 5 (2.78–5.22 Å) respectively. C-H bonds were observed in kaempferol 7-neohesperidoside [Arg383 (3.75 Å)], datiscin [His435 (5.79 Å), Asn (5.46 Å)], afzelin [Pro384 (4.95 Å)], and vitexin [Arg383 (3.81 Å), Gly307 (3.77 Å)].

The aforementioned metabolites engage in numerous hydrophobic interactions at the drug recognition site, facilitated by their ring system and the methyl group on the sugar moiety. Afzelin and diosmin can also form π-cation interactions (with Arg383 and Glu311) and π-alkyl interactions with Arg429 and Ala433 in this hydrophobic region. Their parent structure, guaijaverin (with Arg383) and vitexin (with Glu460), display a comparable interaction pattern.

In this study we found that the reference drug levothyroxine (T4) and triiodothyronine (T3) had the common amino acid such as Arg282, Arg320, Asn331, and His435 on the binding site, which were also found in our docked metabolites like eupatilin. Moreover, three of them were found in quercitrin, isochlorogenic acid C, datiscin, shikimic acid, quinic acid, and vanillic acid.

#### 3.1.2. The ADMET Study

**Figure 2:**
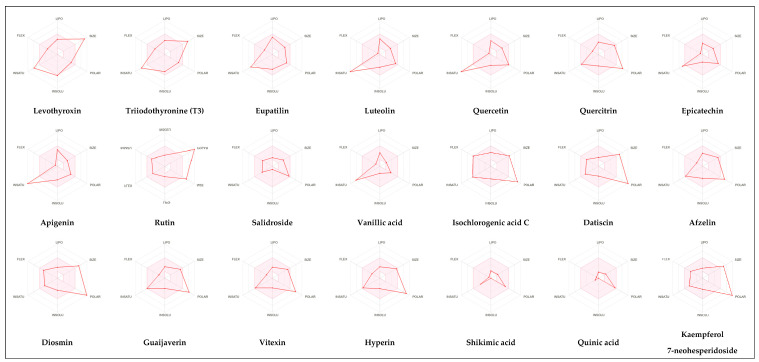
Radar of bioavailability from *H. cordata* ethanolic extract compounds. The pink zone representing ideal lipophilicity, flexibility, polarity, solubility, molecular size, and saturation.

**Figure 3:**
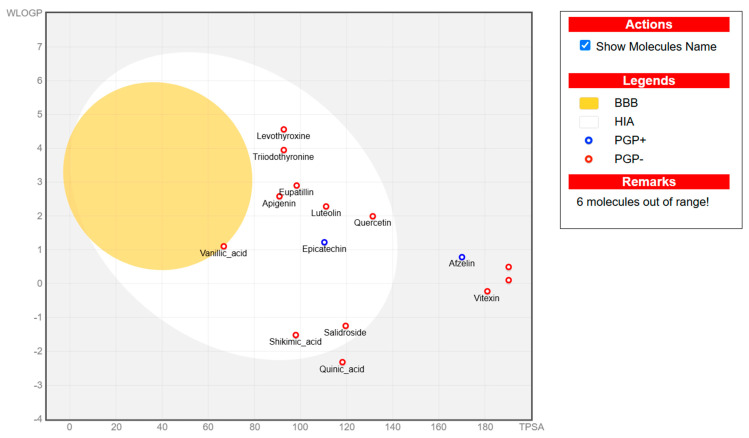
BOILED-EGG model representing the passive gastrointestinal absorption (GIA) and brain penetration of compounds identified from *H. cordata* ethanolic extract. The white region indicates the probability of high GIA, while the yellow region denotes the probability of blood-brain barrier penetration. Molecules that are neither absorbed nor BBB permeant are depicted as out of range. The blue dot represents substrates of P-glycoprotein (Pgp+), whereas the red dot represents non-substrates of P-glycoprotein (Pgp-).

Table 2, Figure 2 and Figure 3 present a summary of the ADME and pharmacokinetic characteristics of the compounds identified in HCEE, analyzed using the SwissADME web tool. Among the isolated compounds, structural information for only 19 was accessible through PubChem. Lipinski’s rules of five (Ro5) posit that a compound’s oral bioavailability is enhanced when it meets the following criteria: molecular weight < 500; fewer than 5 hydrogen bond donors (OH and NH groups); fewer than 10 hydrogen bond acceptors (N and O atoms); fewer than 5 rotatable bonds; and a logP value under 5 [35]. This study demonstrated that eupatilin, luteolin, apigenin, shikimic acid, quinic acid, vanillic acid, quercetin, epicatechin, and salidroside predominately adhered to Lipinski’s (Ro5) criteria.

Absorption and distribution of drugs are influenced by solubility, which is quantified using various measures including LogS, Silicos-IT LogS, ESOL LogS, and Ali LogS. Substances with a LogS value below 6 are considered to have low solubility [36]. The compounds identified in HCEE demonstrated greater solubility compared to traditional anti-hypothyroid medications such as levothyroxine and triiodothyronine (Table 2). Lipophilicity is typically characterized using logP, with molecules generally considered to have high bioavailability when their XlogP3 value falls between −0.7 to +5.0 [37]. The majority of the compounds exhibited logP values within this range, with the exceptions of salidroside, datiscin, shikimic acid, and quinic acid. Oral bioavailability is negatively with the number of rotatable bonds (>10) in molecules and gastrointestinal absorption (GIA) [38]. The majority of the docked metabolites exhibited high oral bioavailability (≥0.55) and GIA due to having fewer than 10 rotatable bonds. However, quercitrin, rutin, neochlorogenic acid, isochlorogenic acid C, datiscin, diosmin, guaijaverin, and hyperin were exceptions to this trend.

To be effective as a pharmaceuical agent, a molecule must reach its target within the body at an adequate concentration and maintain its bioactive form long enough to produce the intended biological effect. In the initial phases of drug discovery, when numerous candidate compounds exist but physical samples are scare, the evaluation of absorption, distribution, metabolism, and excretion (ADME) is increasingly incorporated into drug development. Computational models serve as valid alternatives to experimental methods, with tolls such as Bioavailability Radar, BOILED-Egg, and iLOGP being utilized to predict medicinal chemistry properties pharmacokinetics [36].

The bioavailability radar (Figure 2) illustrates the desirable physicochemical parameters for oral bioavailability, taking into account factors such as size, flexibility, polarity, saturation, solubility, and lipophilicity. The compound’s lipophilicity, indicated as logP, should fall between −0.7 to +5.0. The molecular weight ranges from 150 g/mol to 500 g/mol, while the topological polar surface area (TPSA) should be between 20–130 Ả^2^. The compound’s insolubility, determined using logS (ESOL), should be within 0 to 6. Furthermore, the number of rotatable bonds should range from 0–9, and the unsaturation fraction should be between 0.25 to 1.0, indicating that the fraction of carbon atoms in the sp3 hybridization should not be below 0.25 [39].

The boiled egg model was employed to assess pharmacokinetic properties, evaluating passive gastrointestinal absorption (HIA) and brain penetration (BBB) based on the positioning of molecules in the WLOGP-versus-TPSA referential (Figure 3). The white area signifies a high likelihood of passive absorption by the gastrointestinal tract, while the yellow region (yolk) indicates a high probability of brain penetration. It is noteworthy that the yolk and white regions are not mutually exclusive. The BOILED-Egg model is a valuable tool for screening chemical libraries and supporting drug development. According to this model, eupatillin, luteolin, quercetin, epicatechin, apigenin, salidroside, shikimic acid, and vanillic acid demonstrated high gastrointestinal absorption. Additionally, none of the metabolites crossed the blood-brain barrier (BBB) through lipid-mediated free diffusion, similar to anti-hypothyroid drugs (Figure 3).

### 3.2. Effect of H. cordata Ethanolic Extract on Serum Thyroid Hormones (T3, T4 and TSH)

**Figure 4:**
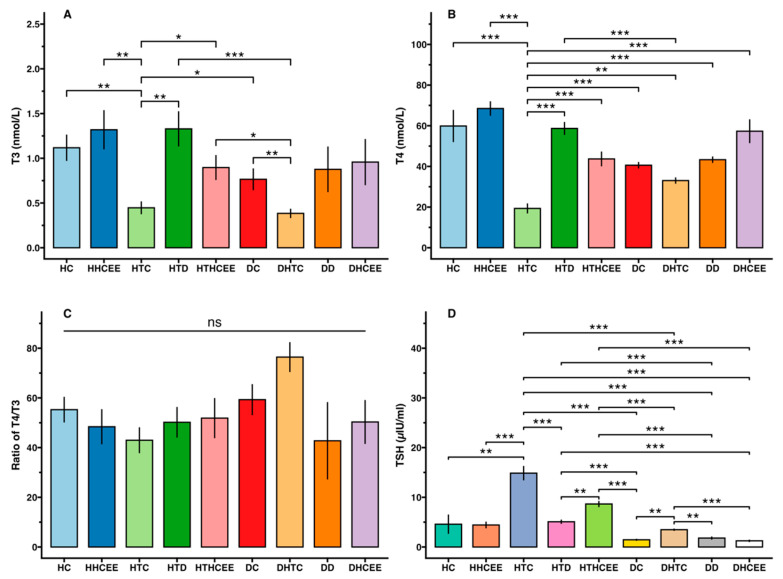
Effect of *H. cordata* ethanolic extract on thyroid hormones among healthy, diabetic, and treated hypothyroid rats. Subfigures label (**A**–**D**) represent T3, T4, ratios of T4/T3, and TSH levels respectively. Values are expressed as mean ± SD, (n = 8). HC (Healthy control); HHCEE (Healthy treated group); HTC (Hypothyroid control); DHTC (Diabetic coupled with hypothyroid control group); HTD (Hypothyroid drug group); HTHCEE (Hypothyroid treated group); DC (Diabetic control); DHCEE (Diabetic treated group); DD (Diabetic drug group); * *p* <0.05, ** *p* <0.01, *** *p* <0.001, ns = non-significant.

**Figure 5:**
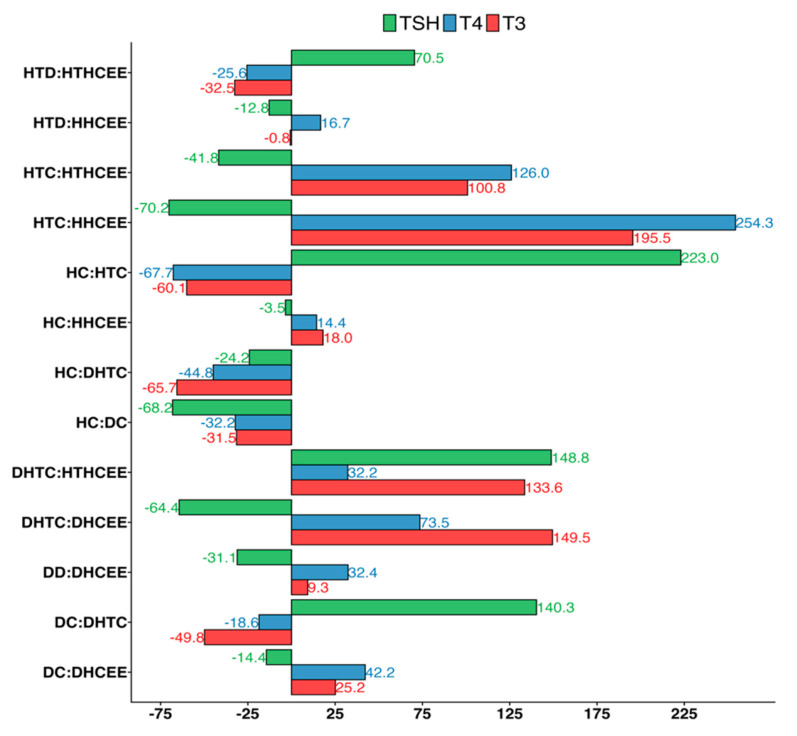
Percentage changes of thyroid hormones among healthy, diabetic, and hypothyroid rats. Here, HC (Healthy control); HHCEE (Healthy treated group); HTC (Hypothyroid control); DHTC (Diabetic coupled with hypothyroid control group); HTD (Hypothyroid drug group); HTHCEE (Hypothyroid treated group); DC (Diabetic control); DHCEE (Diabetic treated group); DD (Diabetic drug group).

In hypothyroid rats (group 2), serum T3 and T4 levels were significantly decreased (*p* < 0.01), with changes of −60.06% and −67.72% respectively. Diabetic rats coupled with hypothyroidism (group 4) also experienced decreased levels of −49.84% and −18.62% (Figure 4 and Figure 5). On the other hand, TSH levels of hypothyroid control (groups 2, and 4) were increased (*p* < 0.001) significantly (223.93% and 140.33% respectively). After treatment with HCEE in both healthy (group 5) and drug induced hypothyroid rats (group 6), serum T3 and T4 levels were increased 18.03% and 100.84%, and 14.38% and 125.96%, respectively in compare with their corresponding control groups (Figure 5). Compared to rats treated with L-thyroxine (group 7), group 6 showed almost identical (slightly reduced) activity. TSH level was reduced significantly (*p* < 0.01; −41.75%) in HCEE treated rats (group 6) as compared to the respective control rats (group 2). The ratio of T4 to T3 in all experimental groups were non-significant (Figure 4).

The present research additionally examined the association between thyroid hormones in healthy and diabetic rats. It was discovered that there was an insignificant negative correlation. The study unveiled that the levels of T3 (−31.53%), T4 (−32.22%) and TSH (−68.44%) were diminished in diabetic rats. Further tests were carried out using the hypothyroidism inducer 6-propyl-2-thiouracil on diabetic rats (group 4). It was observed that the levels of T3 and T4 significantly declined (*p* < 0.001), while TSH levels increased. On the other hand, when rats were treated with HCEE (groups 8 and 6), the opposite results were obtained, which was an increase in T3 (149.51%) and T4 (73.54%) levels and a decrease in TSH (−64.39%) levels. These findings were depicted in Figure 4.

## 4. Discussion

The study conducted an evaluation of the impact of *H. cordata* ethanolic extract (HCEE) on hypothyroidism and diabetes in rats, as well as its potential molecular mechanism. This research represents the first in vivo study to showcase the thyroid-stimulating activity of HCEE in an animal model. In order to identify potential bioactive components in the HCEE responsible for thyroid stimulation, LC-MS/MS analyses were performed, recognizing the crucial role of major components in the biological activity of an extract. Subsequently, the significant compounds of HCEE were subjected to molecular docking studies with thyroid hormone receptor β (TRβ1) (PDB:3GWS). In order for a drug to work well, a strong molecule needs to be present in the body at a high enough concentration to reach its target and stay there in an active form long enough for the expected biological processes to take place [37]. The amount of a dose that reaches the bloodstream without being changed is called bioavailability, and it depends on both metabolism and absorption.

The ADME and pharmacokinetic properties of compounds detected in HCEE were evaluated in this study. It was found that 52.63% of the identified compounds substantially adhered to Lipinski’s rule (Ro5), and 63.18% demonstrated a bioavailability score ≥ 0.55. These results further indicated that 47.39% of the compounds exhibited high gastrointestinal absorption (GIA) and did not cross the blood-brain barrier (BBB) like conventional anti-hypothyroid drugs levothyroxine and triiodothyronine (T3), as evidenced by the ADMET study (Figure 3). Based on the BOILED-Egg model, eupatillin, luteolin, quercetin, epicatechin, apigenin, salidroside, shikimic acid, and vanillic acid exhibited high GIA. Studies have demonstrated a correlation between lipophilicity and toxicity, indicating that substances might bind to hydrophobic protein targets rather than those required, potentially causing harm to biological systems [37]. This study found that logP (standard descriptor for lipophilicity) values for all the detected compounds were between −0.7 and +5, except for salidroside, datiscin, shikimic acid, and quinic acid, suggesting that toxicologically, HCEE appears to be safe. Our target metabolites also behaved like the reference drugs, in particular, their ability in forming hydrophobic and or h-bonds with the catalytic residues of the protein active region, thus presuming their active function activating thyroid functions.

Researchers induced hypothyroidism in rats by administering 0.05% 6-propyl-2-thiouracil (PTU) in drinking water for a period of four weeks. PTU functions by temporarily inhibiting thyroid hormone production through the suppression of crucial enzymes, including thyroperoxidase and peripheral deiodinase [40]. This inhibitory action interferes with the iodination of tyrosyl residues and the coupling of iodotyrosyl residues to form iodothyronine [41,42]. The development of hypothyroidism in the experimental rats was confirmed by asignificant decrease in serum T3 and T4 levels, accompanied by a marked increase in TSH. In contrast, diabetes was induced in rats through the administration of streptozotocin (STZ). For this study, researchers selected rats with fasting blood glucose (FBG) levels exceeding 250 mg/dL (>13.8 mmol/L). The application of HCEE demonstrated promising results in improving thyroid hormone levels in experimentally induced hypothyroidism. Our findings revealed that HCEE treatment resulted in two-fold increase in serum T3, a 2.26-fold increase in T4, and a 1.71-fold reduction in TSH compared to the hypothyroid group. In diabetic rats with hypothyroidism, serum T3, T4, and TSH levels increased by 2.37-fold, 1.32-fold, and 2.49-fold respectively, when compared to their corresponding control groups (Figure 4). However, HCEE treatment in healthy rats (group 5) produced insignificant changes in thyroid hormone levels. The efficacy of HCEE was comparable to that of observed in positive experimental rats (group 9) treated with L-thyroxine (50 mg/kg b.w./day). This study represents the initial investigation into HCEE’s potential to alleviate hypothyroidism. The proposed mechanism suggests that HCEE enhances the expression and/or activity of key enzymes (thyroperoxidase and 5′-deiodinase) essential for thyroid hormone production, thereby stimulating the thyroid gland to secrete these hormones. Additionally, HCEE’s antagonistic effect on PTU may contribute to its effectiveness [24]. Further research is necessary to validate this proposed mechanism, including studies on the expression and activity of thyroperoxidase and 5′-deiodinase in the thyroid gland, as well as the metabolism of PTU following HCEE treatment.

Previous studies have demonstrated that HCEE exhibits potent antioxidant, anti-inflammatory, and free radical scavenging properties, in addition to various pharmacological effects [43]. The present study corroborates these findings, as evidenced by molecular docking studies, which have become essential in contemporary in silico drug development and predict ligand binding affinity to the thyroid receptor protein (TRβ). This methodology can be utilized to identify TRβ-disrupting contaminants, potentially contributing to the understanding of TRβ’s role in thyroid cancer and related disorders. A comprehensive literature review indicated that antioxidant and anti-inflammatory agents may facilitate the repair of PTU-induced thyroid gland damage [44]. Prior research has demonstrated that pentacyclic triterpenes, such as betulinic acid, can ameliorate experimental hypothyroidism by inhibiting NF-kβ activation. NF-kβ initiates T3-dependent induction of 5′-Deiodinase gene expression, resulting in decreased thyroid hormone production [45]. It reduces TSH levels and elevate T3 and T4 levels in PTU-induced hypothyroid rats [46]. Moreover, Chun et al. (2014) and Wang et al. (2023) demonstrated that phytocompounds extracted from *H. chordata* extract can inhibit nuclear factor B (NF-κB) signaling pathways and mitogen-activated protein kinases (MAPKs) [47,48]. These compounds have also been reported to suppress the production of proinflammatory cytokines such as interleukin (IL)-1β, IL-6, tumor necrosis factor (TNF)-α, transforming growth factor (TGF)-β and lipid mediators in the retrobulbar space during the active phase of the thyroid gland [49]. Consequently, it is hypothesized that HCEE, rich in polyphenols and flavonoids (Table 1), may ameliorate experimental hypothyroidism through a comparable mechanism. Singh et al. (2018) posit that the predominance of alpha-adrenergic receptors over beta-adrenergic receptors in hypothyroidism is results in insufficient insulin release from pancreatic β-cells [50]. This leads to a reduced liver glycogen synthesis, intestinal glucose absorption, overall glucose turnover, and peripheral glucose utilization, all of which are positively associated with diabetes [51,52]. Our previous investigation also demonstrated the positive efficacy of HCEE in treating diabetes, dyslipidemia, and associated disorders, including hepatic, cardiac, and renal function markers in experimental rats [32]. Given the absence of previous studies examining the effects of *H. cordata* on hypothyroidism and/or its interaction with diabetes, the precise molecular mechanism underlying its beneficial effects remain undetermined. Further research at the molecular level is necessary to elucidate the specific mechanism by which HCEE influences thyroid function and other biochemical parameters in experimental hypothyroidism and diabetes.

## 5. Conclusions

This study provides preliminary evidence of HCEE’s potential efficacy in addressing hypothyroidism. We hypothesize that its key bioactive components (eupatillin, luteolin, quercetin, quercitrin, epicatechin, apigenin, rutin, salidroside, kaempferol 7-neohesperidoside, isochlorogenic acid C, datiscin, afzelin, diosmin, and guaijaverin) may function as novel thyroid agonists in mitigating hypothyroidism, based on the prediction of molecular docking analysis between HCEE’s metabolites and thyroid hormone receptor. Our results are consistent with the previous study, which demonstrated that phenolic compounds can interact with the drug recognition site of TRH, thus resulting in activated thyroid functions in the hypothyroid-induced mice model. These findings suggest that HCEE may serve as a promising natural alternative to synthetic medications for preventing and reducing thyroid dysfunctions. However, further research is necessary to elucidate the molecular mechanisms and specific active components responsible for HCEE’s potential anti-hypothyroidism effects.

## Figures and Tables

**Table 1:** Characteristics of the binding properties and molecular interactions of bioactive compounds from *H. cordata* ethanolic extract with TRβ1 (3GWS).

Protein	PDB ID	Compounds		Chemical Bond Interaction (Å)			Core Amino Acid on Binding Site
			Binding Energy (kcal/mol)	H-Bonds with Bond Distances (Å) ● Conventional H-Bond ● Carbon H-Bond ● π-Donor H-Bond	Charge ● π-Cation ● π-Anion ● Halogen (Cl, Br, I)	Hydrophobic Interactions ● Alkyl ● π-Sigma ● π-alkyl ● π-Sulfur ● π-π Stacked	
TRβ1	3GWS	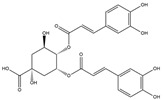 **Isochlorogenic acid C**	−10.0	● Gly344 (1.96), Arg316 (1.89), Arg320 (2.68), Ser314 (3.05)		● Ala317 (4.69), Leu330 (4.24), ● Arg316 (3.78), Ala317 (5.27), Leu346 (4.90) ● Met442 (4.49)	Arg320, Arg282 (VdW), Asn331 (VdW)
		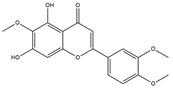 **Eupatilin**	−9.2	● Arg282 (4.46, 5.83), Phe272 (5.16) ● Arg316 (3.93), Asn233 (5.52), Ala279 (4.08) ● Asn331 (5.37)		● Leu330 (4.39), Ile276 (6.11) ● Met313 (4.69) ● Leu346 (5.24), His435 (6.97), Phe455 (6.69), Ile276 (5.60), Arg316 (4.08), Val283 (5.22) ● Ala317 (4.67, 6.31), Leu330 (5.29), Arg316 (6.27)	Arg282, Arg320, Asn331, His435
		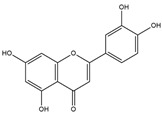 **Luteolin**	−9.1	● Gly344 (4.49), Leu346 (3.48)		● Leu330 (4.48) ● Phe272 (4.52) ● Met442 (9.03) ● Ala317 (7.03), Met313 (5.80), Ile276 (5.90), Leu330 (5.90)	Asn331
		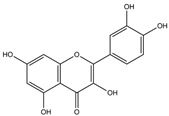 **Quercetin**	−9.0	● Leu346 (3.63), Gly344 (4.17), Leu341 (4.11)		● Leu330 (4.72) ● Phe272 (4.77) ● Ala317 (6.31), Ile276 (6.54), Met313 (5.32), Leu330 (6.16), Leu346 (5.15) ● Met442 (9.00)	Asn331
		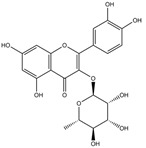 **Quercitrin**	−9.0	● His435 (4.56), Met313 (3.84), Asn331 (3.16) ● Ser314 (3.12)		● Ile276 (5.06, 6.46) ● Ala279 (4.80), Ile276 (4.23) ● Ala317 (6.41), Ile353 (6.64), Leu341 (5.96), Leu330 (5.76), Leu346 (5.73, 4.65)	Arg282, Asn331, His435
		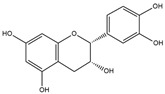 **Epicatechin**	−8.8	● Gly344 (1.98), Asn331 (2.39)		● Ala279 (4.87), Ile276 (5.33), Leu346 (4.99, 5.68), Phe272 (4.65) ● Phe455 (5.99), Phe272 (4.61) ● Leu380 (3.98) ● Met442 (4.90)	Asn331, Arg282
		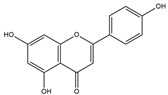 **Apigenin**	−8.8	● Met442 (4.77), Asn331 (4.21) ● Gly345 (3.57)		● Leu330 (4.53) ● Ala317 (6.98), Met313 (5.71), Ile276 (5.89), Leu346 (5.46), Leu330 (5.94) ● Phe272 (4.44)	Arg282, Asn331
		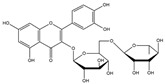 **Rutin**	−8.0	● Phe459 (6.12), Val458 (5.20), Pro384 (2.94, 4.51), Ala436 (3.66)		● Leu440 (4.85) ● Ala436 (5.43), Pro384 (5.33) ● Phe272 (5.70) ● Arg383	
		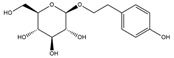 **Salidroside**	−8.0	● Gly344 (3.76), Ile275 (3.68)		● Leu346 (4.82)	His435, Asn331
		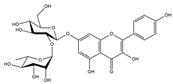 **Kaempferol** **7-neohesperidoside**	−7.9	● Glu311 (4.92), Asp382 (3.93), Gly307 (3.51) ● Arg383 (3.75)		● Arg429 (4.32) ● Pro384 (4.41, 4.78), Ile303 (4.52, 4.86) ● Lys306	His435
		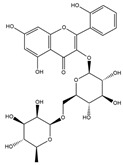 **Datiscin**	−7.9	● Gly344 (4.00) Asn331 (3.91) Ser314 (3.18) ● His435 (5.79), Asn331 (5.46)		● Phe269 (4.74), Phe451 (5.57), Met442 (6.04) ● Phe455 (5.16) ● Leu341 (6.86), Leu330 (5.28, 4.79), Ile353 (6.49), Met313 (3.77), Ala317 (4.55, 3.79, 5.57), Ala279 (6.29, 5.39), Arg316 (5.72) ● Arg320	His435, Arg282, Arg320
		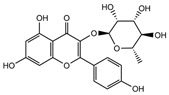 **Afzelin**	−7.8	● Lys306 (3.93), Asp382 (3.71) ● Pro384 (4.95)	● Arg383 (4.01, 5.32), Glu311 (7.64)	● Met310 (4.91) ● Arg429 (6.11), Pro384 (4.69)	His435
		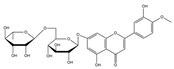 **Diosmin**	−7.8	● Phe459 (4.11)	● Arg383 (4.69), Glu311 (7.96, 7.96)	● Arg429 (5.41), Met430 (5.75), Ala436 (4.04), Leu440 (4.47) ● Ala433 (5.42, 6.72), Arg429 (5.18) ● Lys306	
		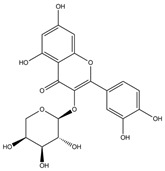 **Guaijaverin**	−7.8	● Val458 (5.94)	● Arg383 (4.19, 5.39)	● Pro384 (4.64) ● Ala433 (5.32), Arg429 (6.29), Gly307 (4.73)	His435
		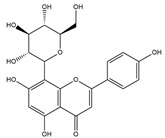 **Vitexin**	−7.5	● Glu311 (5.22), Ile303 (4.28), Gly307 (3.77), Pro384 (3.37), Ala436 (2.78) ● Arg383 (3.81), Gly307 (3.69)	● Glu460 (5.68, 6.64)	● Ala436 (5.80), Pro384 (5.49)	-
		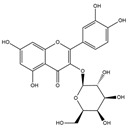 **Hyperin**	−6.2	● Gly344 (4.62), Asn331 (3.34), Ser314 (2.87)		● Met442 (7.49) ● Phe272 (4.92), Phe455 (7.07) ● Leu346 (7.21), Ala279 (6.96), Ile276 (3.84, 4.39, 5.98), Leu330 (5.43), Ile275 (5.77)	Asn331
		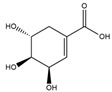 **Shikimic acid**	−6.2	● Arg320 (4.10, 4.34), Asn331 (4.10, 4.21), Thr329 (4.20) ● Met313 (3.93), Asn331 (4.87)			Arg282, Arg320, Asn331
		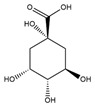 **Quinic acid**	−6.0	● Arg316 (4.91), Met313 (4.80)		● Asn331, Arg282	Arg282, Asn331, His435
		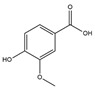 **Vanillic acid**	−5.2	● Arg282 (4.58) ● Ala279 (3.87), Asn (4.83)		● Val283 (5.25), Met313 (4.06) ● Ala279 (4.41), Ala317 (5.86), Arg316 (4.73), Leu330 (6.32) ● Met313 (4.79)	Arg282, Arg320, Asn331
		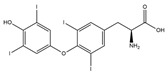 **Levothyroxine (T4)**	−7.3		● Phe272 (4.92), Gly344 (4.80)	● Leu330 (4.48), Leu346 (5.33) ● Phe459 (6.17), Phe455 (6.01), Met310 (4.56), His435 (5.67), Ile275 (6.08), Ile276 (5.37, 4.46), Leu330 (4.87, 5.25), Ile353 (4.92) ● Ile276 (4.78), Ala279 (5.46), Leu341 (5.62), Ala317 (5.25), Met313 (4.56, 5.65) ● Asn331, Arg282	Arg282, Arg320, Asn331, His435
		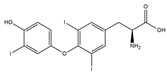 **Triiodothyronine (T3)**	−9.0	● Arg282 (4.91) ● Arg316 (3.96)	● Phe272 (4.88), Gly344 (5.07)	● Leu330 (4.79), Leu346 (5.33) ● Met310 (7.09) ● Ile276 (6.07), Ala317 (5.47), Met313 (4.28), Ala279 (5.26) ● Asn331	Arg282, Arg320, Asn331, His435

**Table 2:** Physicochemical, lipophilicity, solubility, and pharmacokinetic properties of *H. cordata* ethanolic extract.

Properties	Physicochemical Properties					Lipophilicity		Water Solubility						Pharmacokinetics		Druglikeness	
	Molecular wt. (g/mol)	Rotatable Bond	H-Bond Acceptors	H-Bond Donors	TPSA (Ả2)	XlogP3	WLogP	ESOL LogS	ESOL Class	Ali LogS	Ali Class	Silicos-IT LogS	Silicos-IT Class	GI Absorption	Blood Brain Barrier	Lipinski’s Violation	Bioavailability Score
Levothyroxin	776.87	5	5	3	92.78	2.36	4.56	−6.18	PS	−3.95	S	−6.79	PS	High	No	Yes; 1	0.55
Triiodothyronine (T3)	650.97	5	5	3	92.78	1.71	3.95	−5.01	MS	−3.27	S	−6.03	PS	High	No	Yes; 1	0.55
Eupatilin	344.32	4	7	2	98.36	3.40	2.90	−4.33	MS	−5.14	MS	−5.33	MS	High	No	Yes; 0	0.55
Luteolin	286.24	1	6	4	111.13	2.53	2.28	−3.71	S	−4.51	MS	−3.82	S	High	No	Yes; 0	0.55
Quercetin	302.24	1	7	5	131.36	1.54	1.99	−3.16	S	−3.91	S	−3.24	S	High	No	Yes; 0	0.55
Quercitrin	448.38	3	11	7	190.28	0.86	0.49	−3.33	S	−4.44	MS	−2.08	S	Low	No	No; 2	0.17
Epicatechin	290.27	1	6	5	110.38	0.36	1.22	−2.22	S	−2.24	S	−2.14	S	High	No	Yes; 0	0.55
Apigenin	270.24	1	5	3	90.90	3.02	2.58	−3.94	S	−4.59	MS	−4.40	MS	High	No	Yes; 0	0.55
Rutin	610.52	6	16	10	269.43	−0.33	−1.69	−3.30	S	−4.87	MS	−0.29	S	Low	No	No; 3	0.17
Salidroside	300.30	5	7	5	119.61	−1.05	−1.25	−0.92	VS	−0.97	VS	−0.44	S	High	No	Yes; 0	0.55
Isochlorogenic acid C	516.45	9	12	7	211.28	1.52	0.81	−3.65	S	−5.57	MS	−1.16	S	Low	No	No; 3	0.11
Datiscin	594.52	6	15	9	249.20	−0.93	−1.39	−2.83	S	−3.82	S	−0.88	S	Low	No	No; 3	0.17
Afzelin	432.38	3	10	6	170.05	1.22	0.78	−3.47	S	−4.39	MS	−2.67	S	Low	No	Yes; 1	0.55
Diosmin	608.54	7	15	8	238.20	0.14	−1.09	−3.51	S	−4.70	MS	−1.57	S	Low	No	No; 3	0.17
Guaijaverin	434.35	3	11	7	190.28	0.43	0.10	−2.99	S	−3.99	S	−1.94	S	Low	No	No; 2	0.17
Vitexin	432.38	3	10	7	181.05	0.21	−0.23	−2.84	S	−3.57	S	−2.38	S	Low	No	Yes; 1	0.55
Hyperin	464.38	4	12	8	210.51	0.36	−0.54	−3.04	S	−4.35	MS	−1.51	S	Low	No	No; 2	0.17
Shikimic acid	174.15	1	5	4	97.99	−1.72	−1.52	0.23	HS	0.18	HS	1.75	S	High	No	Yes; 0	0.56
Quinic acid	192.17	1	6	5	118.22	−2.37	−2.32	0.53	HS	0.43	HS	2.08	S	Low	No	Yes; 0	0.56
Vanillic acid	168.15	2	4	2	66.76	1.43	1.10	−2.02	S	−2.44	S	−1.32	S	High	No	Yes; 0	0.85
Kaempferol 7-neohesperidoside	594.52	6	15	9	249.20	−0.31	−1.39	−3.22	S	−4.46	MS	−0.88	S	Low	No	No; 3	0.17

Here, PS: poorly soluble; MS: moderately soluble; S: soluble; VS: very soluble; HS: highly soluble.

## Data Availability

All data generated or analyzed during this study are included in this manuscript.
